# Supplementary material for: Core and rod structures of a thermophilic cyanobacterial light-harvesting phycobilisome
Source: Nat Commun. 2022 Jun 17;13:3389. doi: 10.1038/s41467-022-30962-9 (PMC9205905; doi:10.1038/s41467-022-30962-9)
Supplement: Supplementary file 1 — Supplementary Information [file 41467_2022_30962_MOESM1_ESM.pdf]

Supplementary Information

Core and rod structures of a thermophilic cyanobacterial light-harvesting phycobilisome

Keisuke Kawakami[*‡], Tasuku Hamaguchi[*], Yuu Hirose, Daisuke Kosumi, Makoto Miyata, Nobuo Kamiya, Koji Yonekura[‡]

[*]These authors contributed equally to this work.
[‡]Corresponding author. Email: kawakami.k@spring8.or.jp (KK), yone@spring8.or.jp (KY)

This file includes:

Supplementary Figures 1–16

Supplementary Tables 1–7

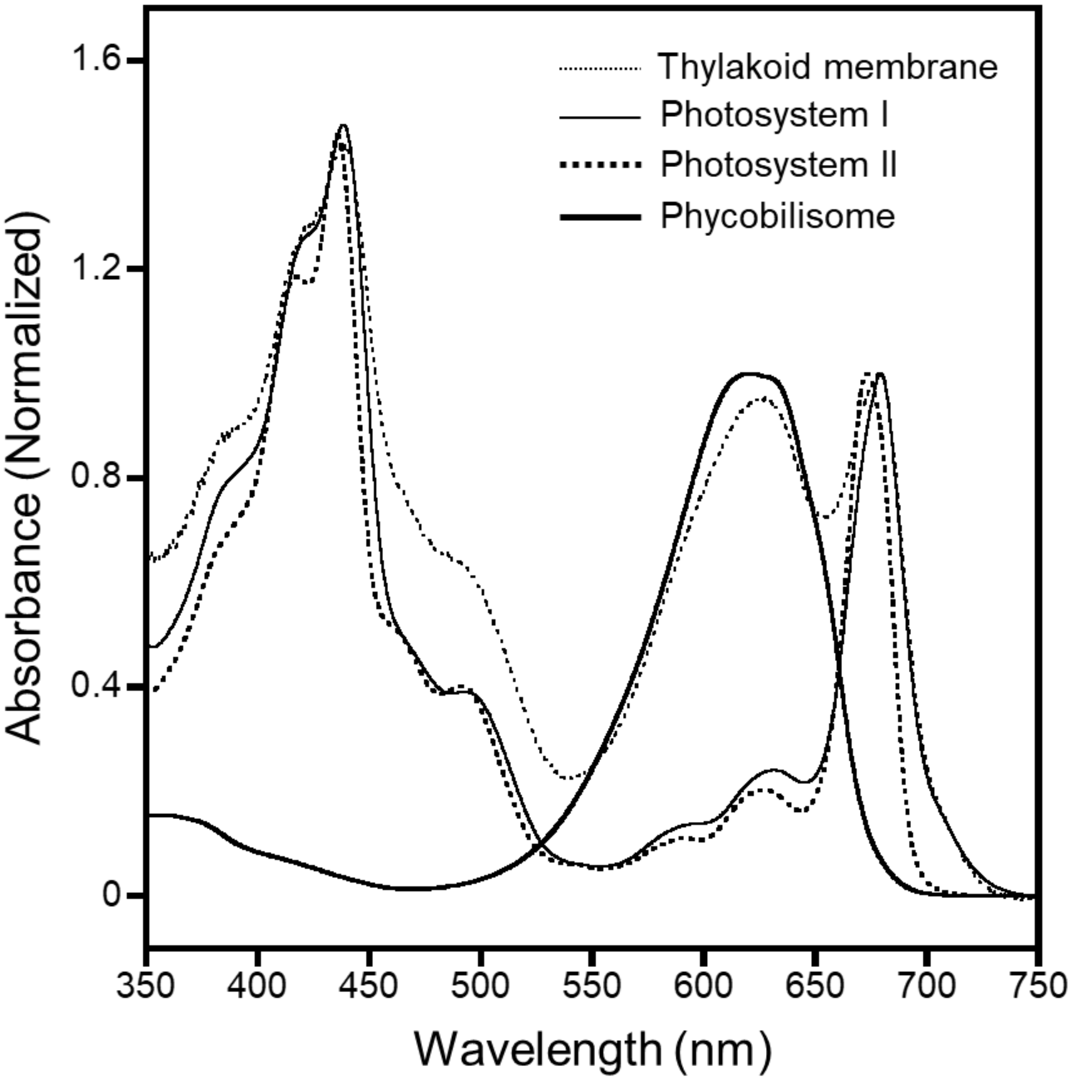

Supplementary Figure 1. Absorption spectra of photosynthetic protein complexes.

Thylakoid membrane, thin dotted line; photosystem I, thin solid line; photosystem II, bold dotted line; phycobilisome, bold solid line.

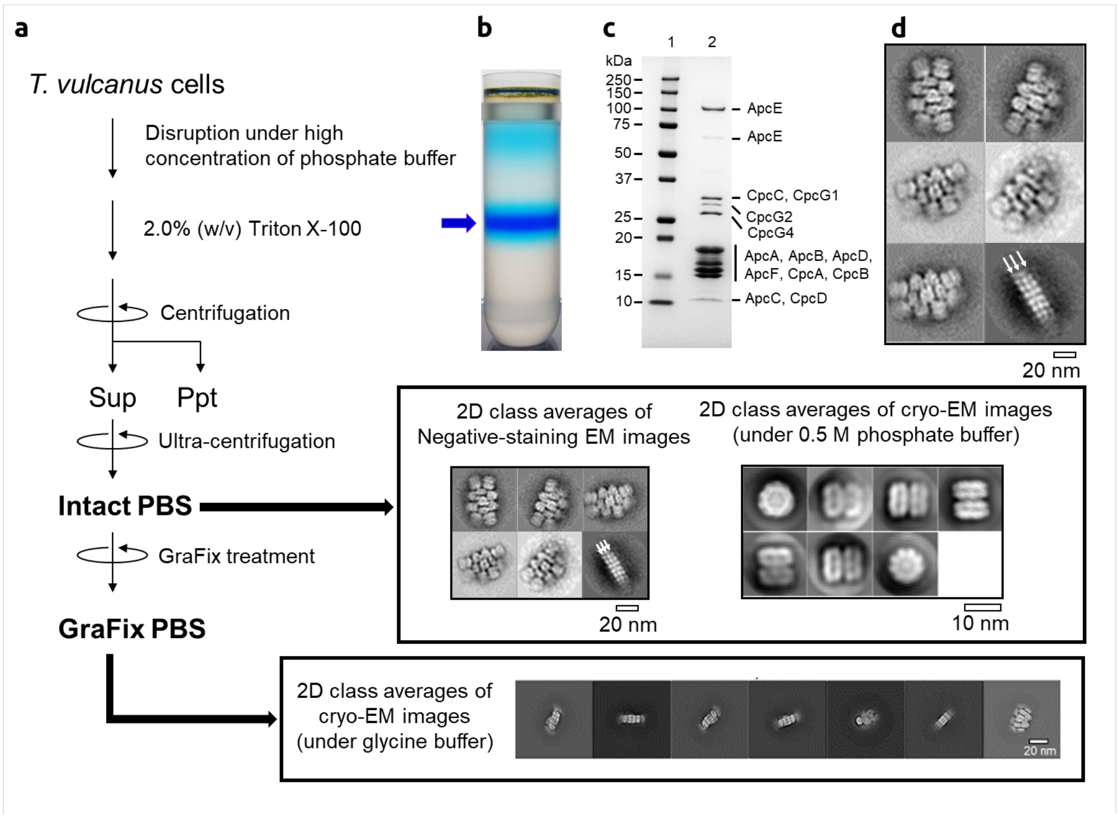

Supplementary Figure 2. Sample preparation and polypeptide analysis. (a) Flowchart of sample preparation and cryo-EM analysis of GraFix PBS and PC rod. **(b)** The intact PBS (blue arrow) prepared by ultra-centrifugation. **(c)** Polypeptide analysis: lane 1, marker; lane 2, the prepared intact PBS. This experiment was repeated more than three times independently to confirm the reproducibility of the sample preparation. **(d)** 2D class averages of negative-staining EM images of the intact PBS. Magnified views in (a). White arrows in the 2D class averages of the prepared intact PBS indicate the number of APC trimers in the A (A') cylinder.

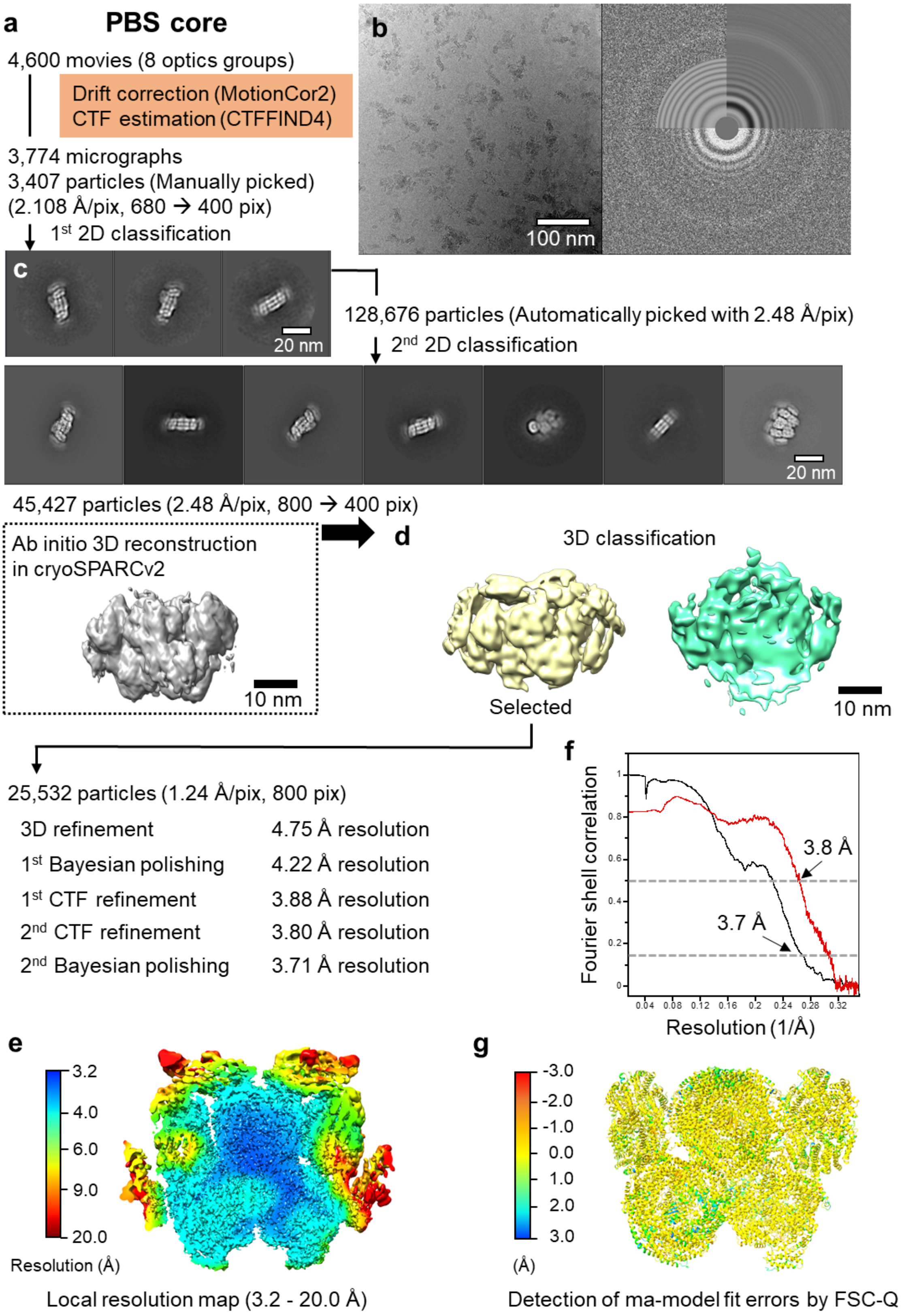
a
PBS core
4,600 movies (8 optics groups)
Drift correction (MotionCor2)
CTF estimation (CTFFIND4)
3,774 micrographs
3,407 particles (Manually picked)
(2.108 Å/pix, 680 → 400 pix)
1st 2D classification
b
100 nm
c
20 nm
128,676 particles (Automatically picked with 2.48 Å/pix)
2nd 2D classification
20 nm
45,427 particles (2.48 Å/pix, 800 → 400 pix)
Ab initio 3D reconstruction
in cryoSPARCv2
10 nm
d
3D classification
Selected
10 nm
25,532 particles (1.24 Å/pix, 800 pix)
3D refinement 4.75 Å resolution
1st Bayesian polishing 4.22 Å resolution
1st CTF refinement 3.88 Å resolution
2nd CTF refinement 3.80 Å resolution
2nd Bayesian polishing 3.71 Å resolution
f
Fourier shell correlation
3.8 Å
3.7 Å
Resolution (1/Å)
e
3.2
4.0
6.0
9.0
20.0
Resolution (Å)
Local resolution map (3.2 - 20.0 Å)
g
-3.0
-2.0
-1.0
0.0
1.0
2.0
3.0
(Å)
Detection of ma-model fit errors by FSC-Q

Supplementary Figure 3. Data collection and image processing workflow of the PBS core using cryo-EM. (a) Overview of the data processing workflow. The resolution was estimated based on the gold standard Fourier shell correlation (FSC) criteria of 0.143. **(b)** Representative micrograph and Thon ring. The homogeneity of the PBS core particles was evaluated from 4,600 images, and the images were used for data processing. **(c)** Good reference-free 2D class of the PBS core. **(d)** 3D classification of the PBS core. **(e)** A slice through the local resolution map of the PBS core to show the internal details. **(f)** FSC curves for 3D reconstruction of the cryo-EM map and the refined model versus the overall 3.7 Å map. Black, gold-standard curve with a value of 0.143 at 3.7 Å resolution; red, FSC curve calculated between the cryo-EM map and the refined structure model of the PBS core. The map-model FSC has a value of 0.5 at 3.8 Å resolution. **(g)** FSC-Q values calculated for the PBS core structural model. Atoms with FSC-Q values close to zero mean that the structural model is supported by the two half maps signal, while FSC-Q values that are positively away from zero corresponded to areas where the fit between the model and the map is low, or where the resolution of the map is low. On the other hand, negative FSC-Q values correspond to the atoms being correlated with noise, which means overfitting. Based on the FSC-Q validation, it

can be assessed that the refined PBS core model is almost free of overfitting and that the model is reasonable for the cryo-EM map at 3.7 Å resolution.

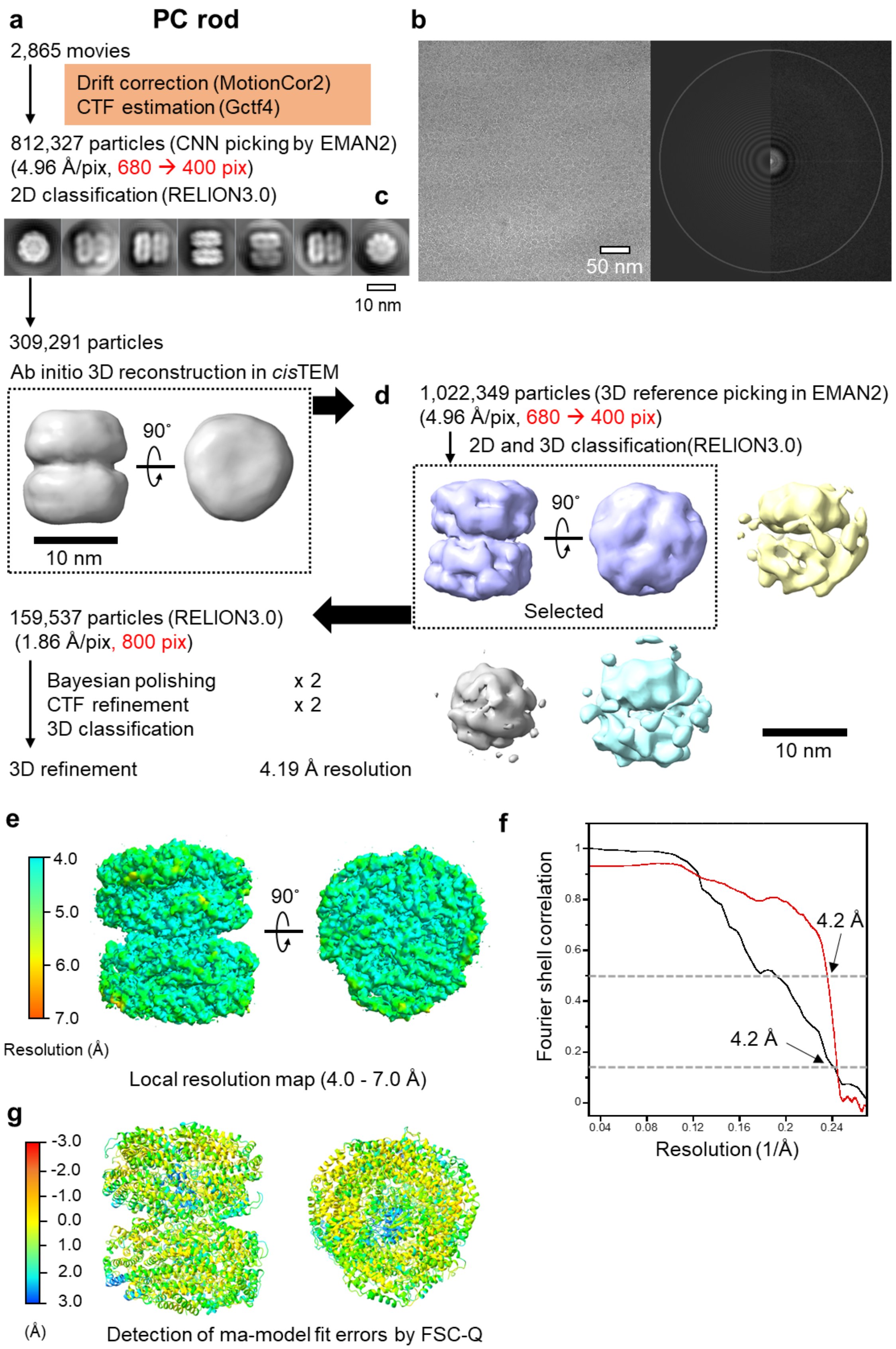
a
PC rod
2,865 movies
Drift correction (MotionCor2)
CTF estimation (Gctf4)
812,327 particles (CNN picking by EMAN2)
(4.96 Å/pix, 680 → 400 pix)
2D classification (RELION3.0)
c
10 nm
309,291 particles
Ab initio 3D reconstruction in cisTEM
90°
10 nm
b
50 nm
d
1,022,349 particles (3D reference picking in EMAN2)
(4.96 Å/pix, 680 → 400 pix)
2D and 3D classification(RELION3.0)
90°
Selected
10 nm
159,537 particles (RELION3.0)
(1.86 Å/pix, 800 pix)
Bayesian polishing x 2
CTF refinement x 2
3D classification
3D refinement
4.19 Å resolution
e
4.0
5.0
6.0
7.0
Resolution (Å)
90°
Local resolution map (4.0 - 7.0 Å)
f
Fourier shell correlation
Resolution (1/Å)
4.2 Å
4.2 Å
g
-3.0
-2.0
-1.0
0.0
1.0
2.0
3.0
(Å)
Detection of ma-model fit errors by FSC-Q

Supplementary Figure 4. Data collection and image processing workflow of the PC rod by cryo-EM. (a) Overview of the data processing workflow. The resolution was estimated based on the gold standard Fourier shell correlation (FSC) criteria of 0.143. **(b)** Representative micrograph and Thon ring. The homogeneity of the PC rod particles was evaluated from 2,865 images, and the images were used for data processing. **(c)** Good reference-free 2D class of the PC rod. **(d)** 3D classification of the PBS core. **(e)** The local resolution map of the PC rod. **(f)** FSC curves for 3D reconstruction of the cryo-EM map and the refined model versus the overall 4.2 Å map. Black, gold-standard curve with a value of 0.143 at 4.2 Å resolution; red, FSC curve calculated between the cryo-EM map and the refined structure model of the PC rod. The map-model FSC has a value of 0.5 at 4.2 Å resolution. **(g)** FSC-Q values calculated for the PC rod structural model. Atoms with FSC-Q values close to zero mean that the structural model is supported by the two half maps signal, while FSC-Q values that are positively away from zero corresponded to areas where the fit between the model and the map is low, or where the resolution of the map is low. In almost all cases for resolutions worse than 4 Å, the FSC-Q values of a structural model increase to >1.5 Å[67]. On the other hand, negative FSC-Q values correspond to the atoms being correlated with noise, which means overfitting. Based on the FSC-Q validation, it can be assessed that the refined PC

rod model is almost free of overfitting and that the model is reasonable for the cryo-EM map at 4.2 Å resolution.

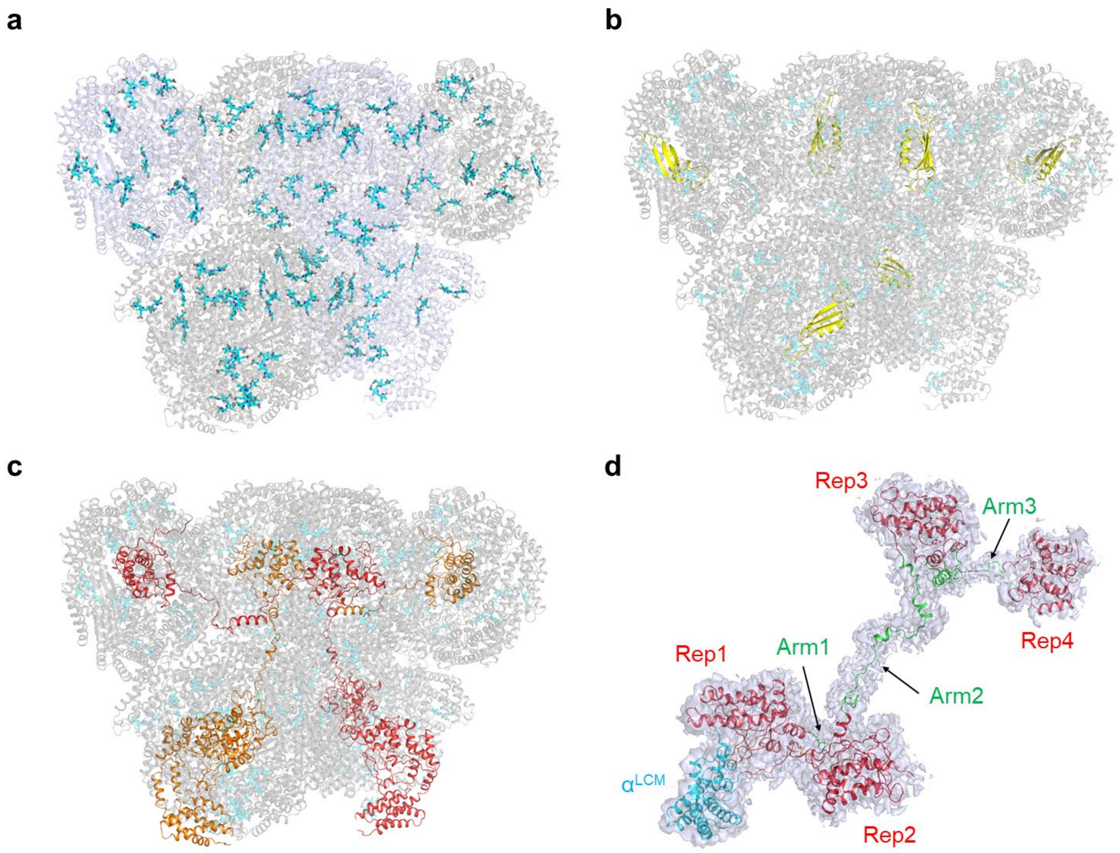

Supplementary Figure 5. Arrangement of chromophores and linker proteins in the PBS core from *T. vulcanus*. (a) Phycocyanobilins (cyan) distribution in the PBS core. **(b)** Arrangement of six ApcCs (L_C, yellow) in the PBS core. **(c)** Arrangement of two ApcEs (L_{CM}; orange and red) in the PBS core. **(d)** Cryo-EM map of ApcE (L_{CM}) and its refined model. ApcE is composed of the α^{LCM} (cyan), Reg1–4 (red), and Arm1–3 (green). Cryo-EM map of ApcE is shown in a surface representation at 1.0 sigma contour levels.

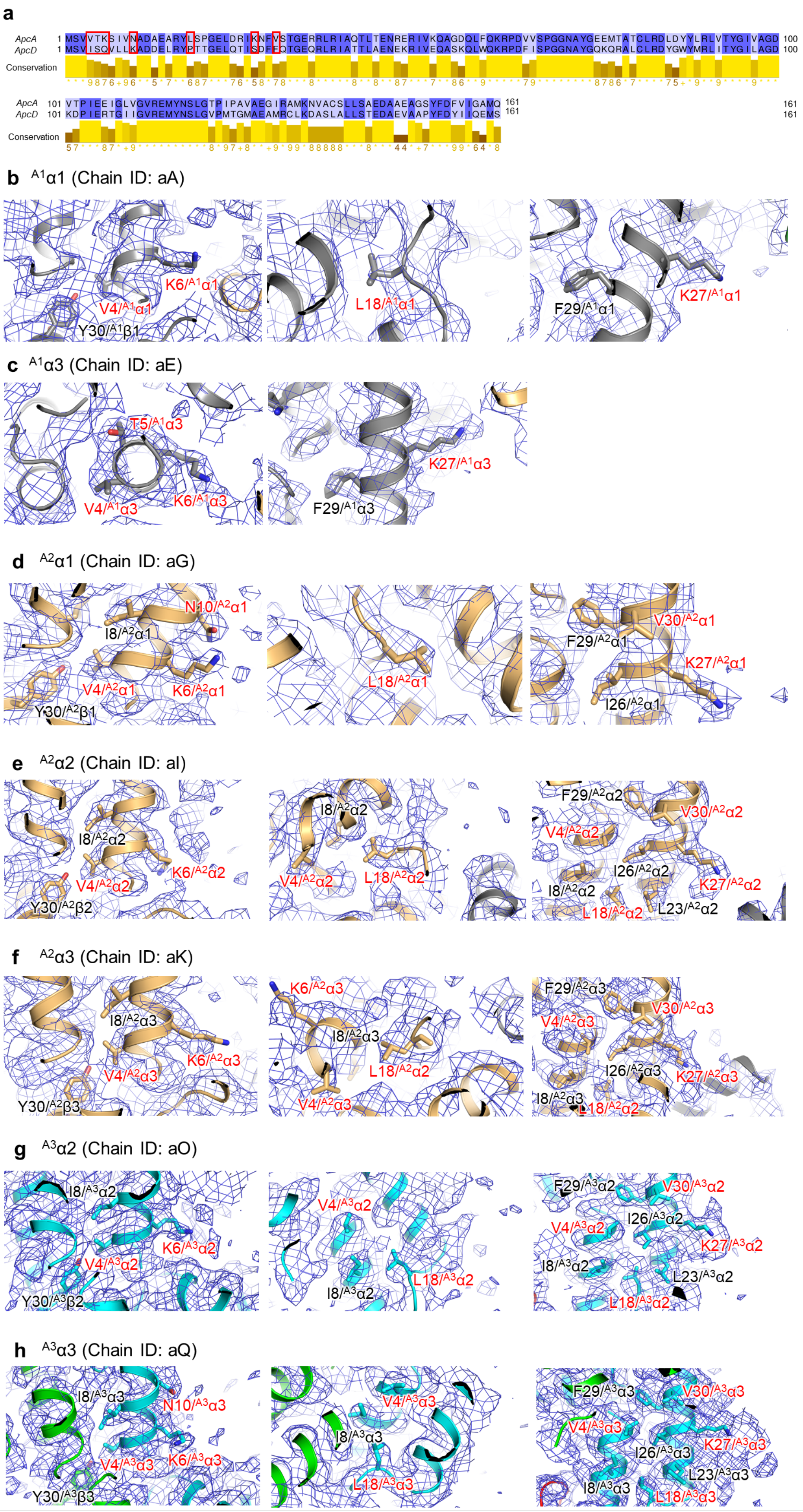
a
ApcA 1 MSVVTKSIVNADAEARYLSPGELDRIKNFVSTGERRLRIAQTLTENRERIVKQAGDQLFQKRPDVVSPGGNAYGEEMTATCLRDLDYYLRLVTYGIVAGD 100
ApcD 1 MSVISQVLLKADDELRYPTTGELQTISDFFQTGEQRLRIATTLAENEKRIVEQASKQLWQKRPDFISPGGNAYGQKQRALCLRDYGWYMRLITYGILAGD 100
Conservation
ApcA 101 VTPIEEIGLVGVREMYNSLGTPIPAVAEGIRAMKNVACSLLSAEDAAEAGSYFDFVIGAMQ 161
ApcD 101 KDPIERTGIIGVREMYNSLGVPMTGMAEAMRCLKDASLALLSTEDAEVAAPYFDYIIQEMS 161
Conservation
b A1α1 (Chain ID: aA)
K6/A1α1
V4/A1α1
Y30/A1β1
L18/A1α1
F29/A1α1
K27/A1α1
c A1α3 (Chain ID: aE)
T5/A1α3
V4/A1α3
K6/A1α3
K27/A1α3
F29/A1α3
d A2α1 (Chain ID: aG)
N10/A2α1
I8/A2α1
V4/A2α1
K6/A2α1
Y30/A2β1
L18/A2α1
V30/A2α1
F29/A2α1
K27/A2α1
I26/A2α1
e A2α2 (Chain ID: aI)
I8/A2α2
V4/A2α2
K6/A2α2
Y30/A2β2
L18/A2α2
F29/A2α2
V30/A2α2
I26/A2α2
K27/A2α2
L23/A2α2
f A2α3 (Chain ID: aK)
I8/A2α3
K6/A2α3
V4/A2α3
Y30/A2β3
L18/A2α2
F29/A2α3
V30/A2α3
I26/A2α3
K27/A2α3
g A3α2 (Chain ID: aO)
I8/A3α2
K6/A3α2
V4/A3α2
Y30/A3β2
L18/A3α2
F29/A3α2
V30/A3α2
I26/A3α2
K27/A3α2
L23/A3α2
h A3α3 (Chain ID: aQ)
I8/A3α3
N10/A3α3
V4/A3α3
K6/A3α3
Y30/A3β3
L18/A3α3
F29/A3α3
V30/A3α3
I26/A3α3
K27/A3α3
L23/A3α3

Supplementary Figure 6. Identification of ApcD in the PBS core. In this study, the subunit in the PBS core that could not be identified as ApcA (i.e., $^{A1}\alpha 2$, Chain ID: aC/dC) was identified as ApcD. **(a)** Amino acid sequence alignment of ApcA and ApcD. The colors of the amino acid residues are represented in percentage identities using Jalview. The amino acid residues in the red boxes are residues that differ between ApcA and AcpD. **(b-h)** Each cryo-EM map in α subunit in the PBS core is shown in a mesh representation at 1.0–3.0 sigma contour levels.

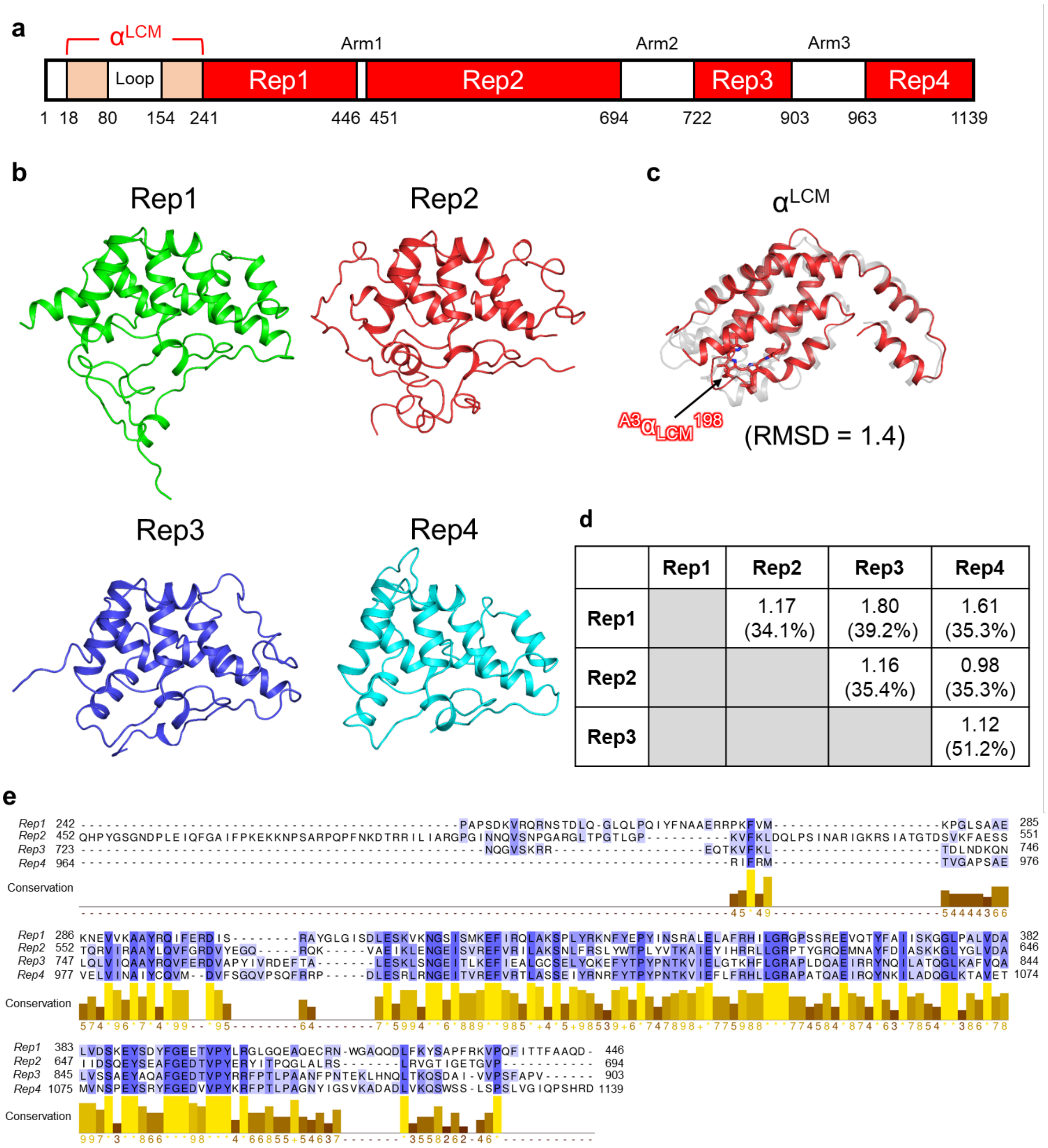

	Rep1	Rep2	Rep3	Rep4
Rep1		1.17 (34.1%)	1.80 (39.2%)	1.61 (35.3%)
Rep2			1.16 (35.4%)	0.98 (35.3%)
Rep3				1.12 (51.2%)

Supplementary Figure 7. Structure of α^LCM, Rep1–4 of ApcE (L_CM). (a) Diagram of the structural element of ApcE. **(b)** Structures of Reg1–4. Reg1, green; Reg2, red; Reg3, blue; Reg4, cyan. **(c)** Superposition with α^LCM and ApcA. α^LCM, red; transparent gray, crystal structure of APC (PDB code: 3DBJ). The value in parenthesis indicates the root

mean square deviations (RMSD). Secondary structure matching in CCP4 was used to calculate the value of RMSD. **(d)** The values indicate RMSD. The values in parentheses indicate sequence identities. **(e)** Amino acid sequence alignment of the Rep1–4 of L_{CM}. The colors of the amino acid residues are indicated by percentage identities using Jalview.

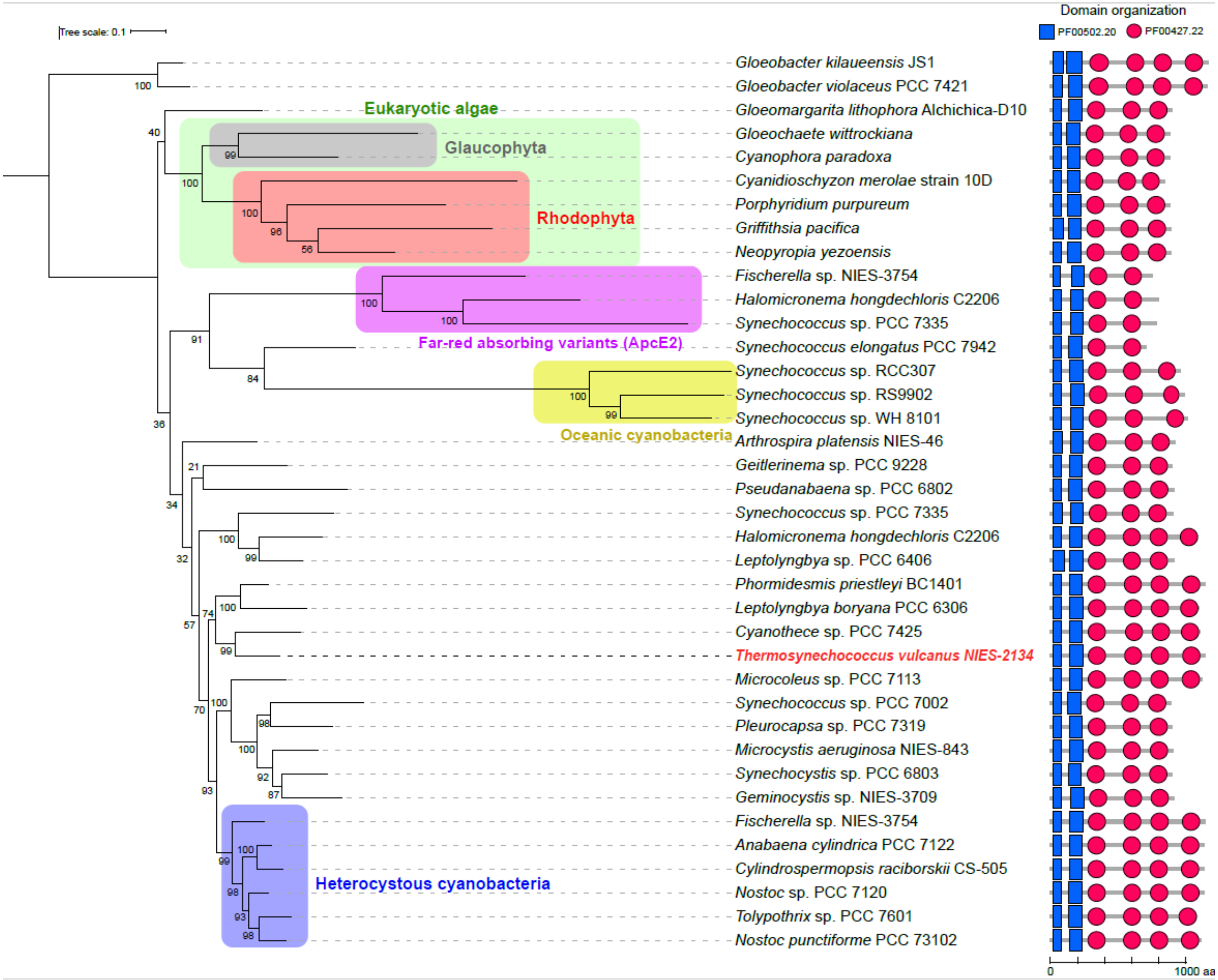

Supplementary Figure 8. Phylogenic analysis of ApcE (L_{CM}) subunit.

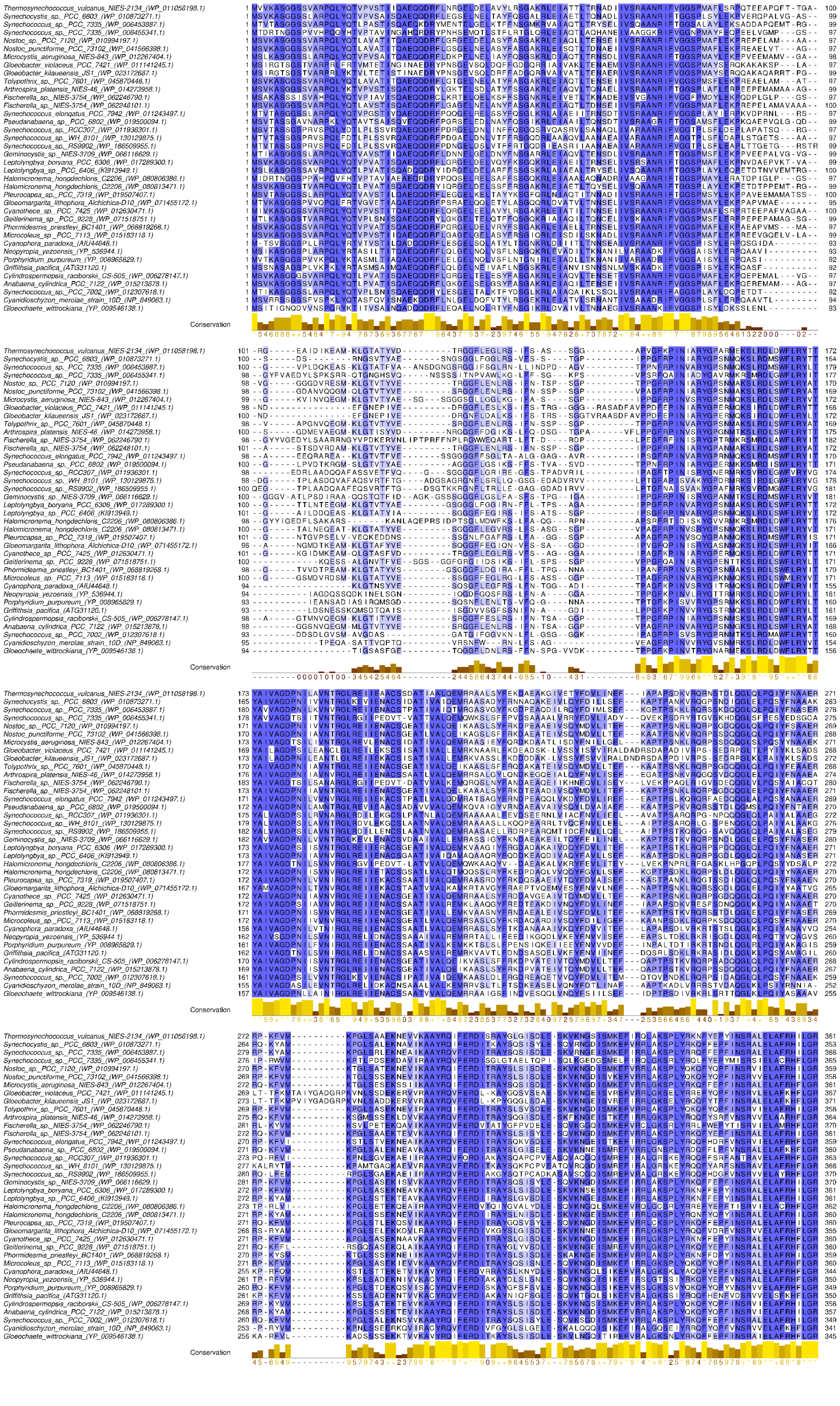
Conservation

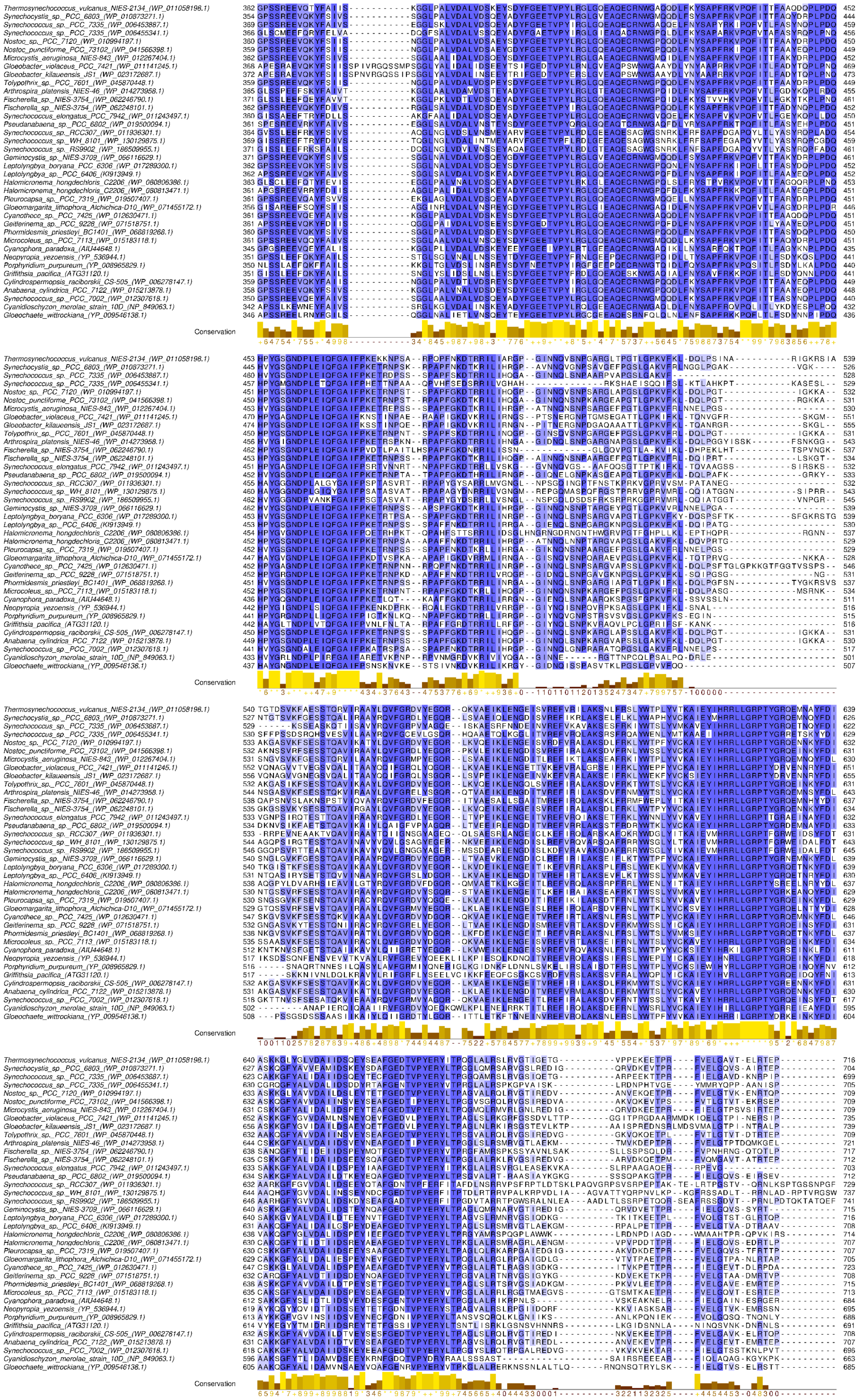

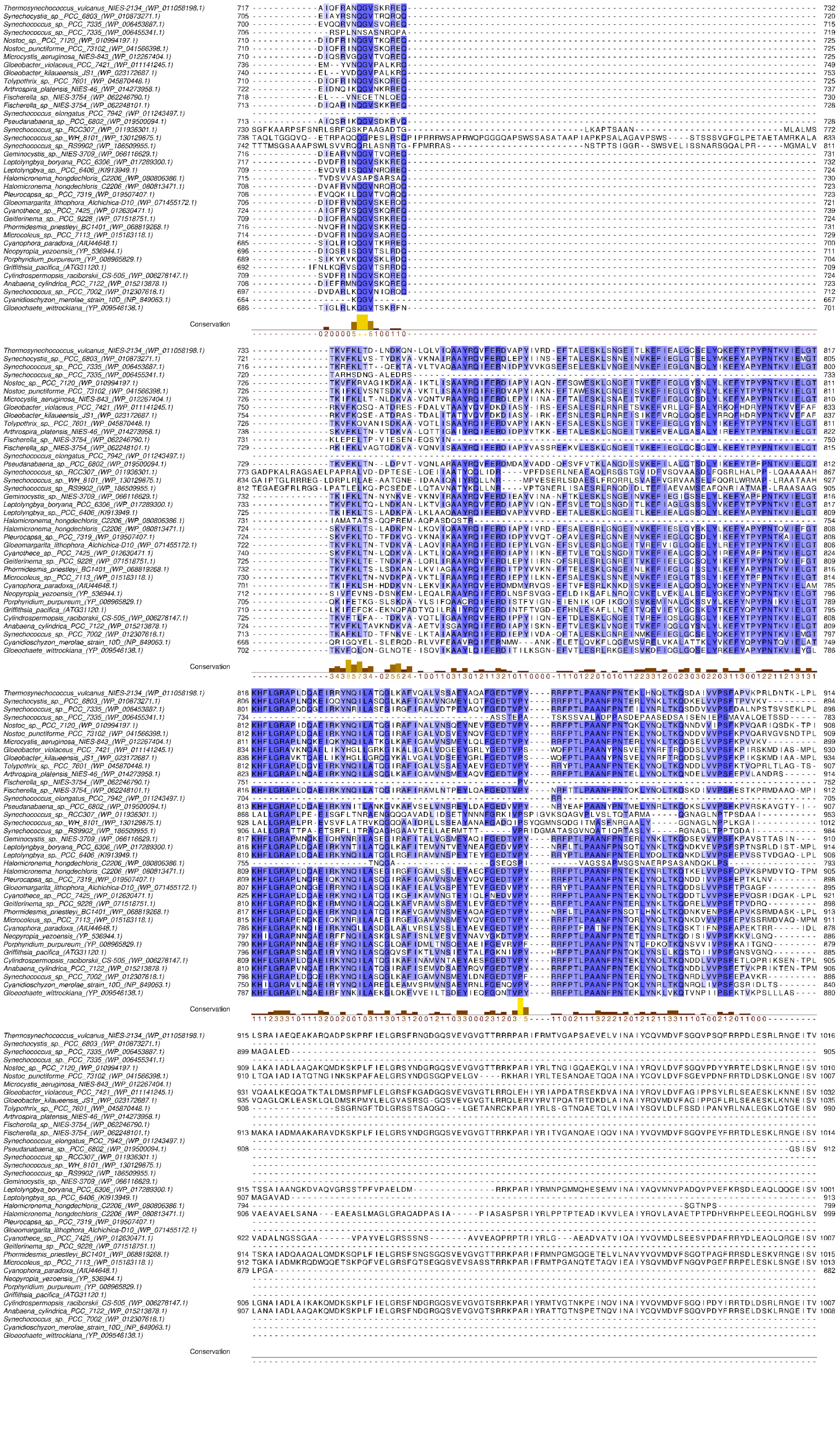

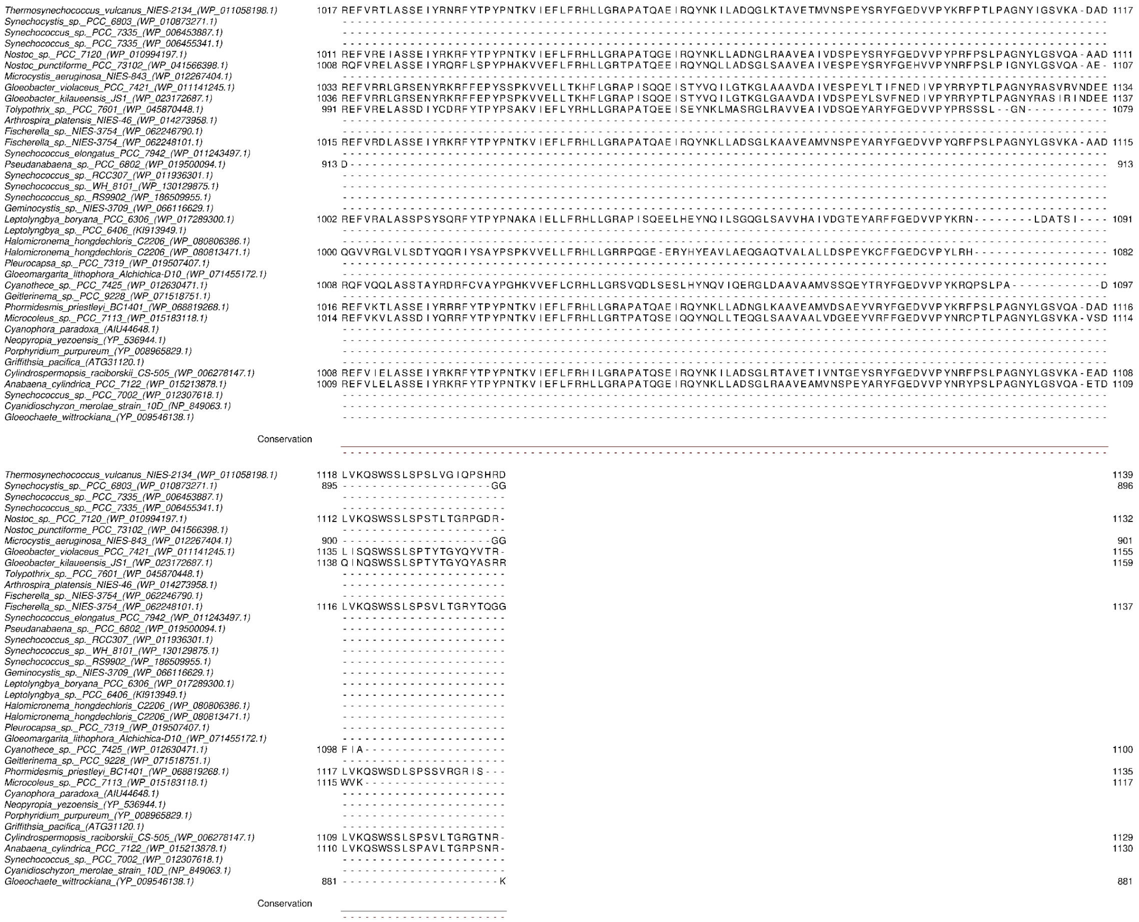

Supplementary Figure 9. Amino acid sequence alignment of ApcE (L_{CM}) subunit from selected strains of cyanobacteria, glaucophytes, and rhodophytes. The colors of the amino acid residues are indicated by percentage identities using Jalview.

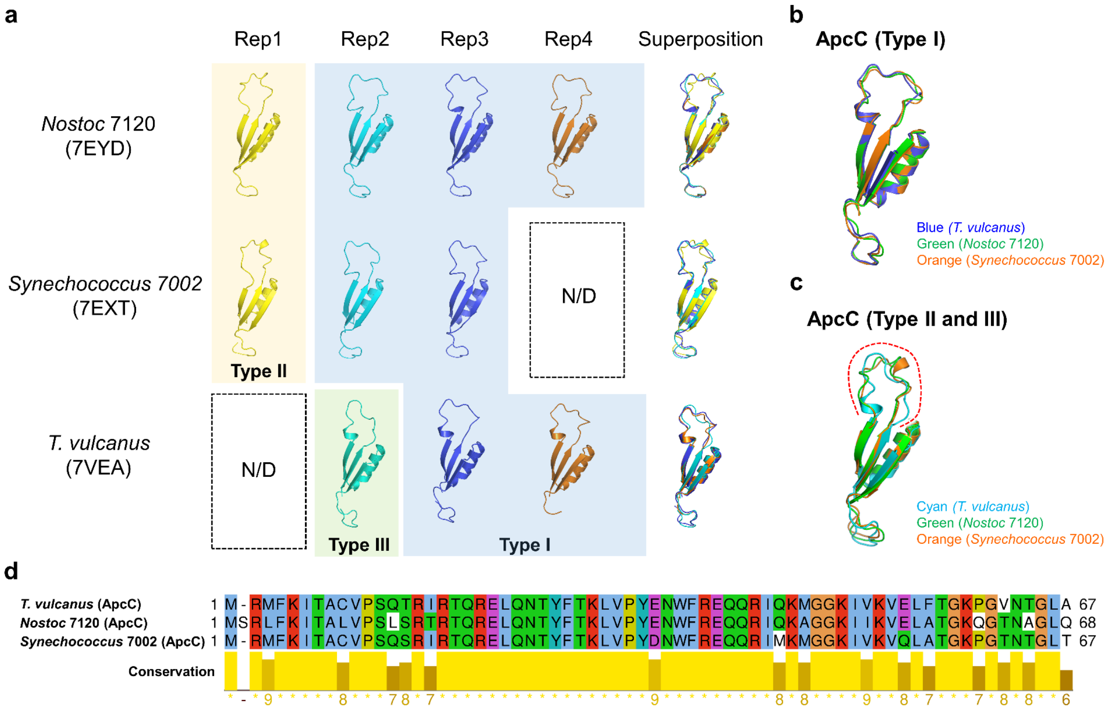

Supplementary Data Figure 10. Structure of ApcC interacting with repetitive domain (Rep) of ApcE (LCM) in different cyanobacteria. (a) Structures of ApcC interacting with each Rep. N/D: Not detected. **(b)** Superposition with ApcCs (Type I) of each cyanobacterium. Blue, ApcC interacting with Rep3 of *T. vulcanus*; green, ApcC interacting with Rep2 of *Nostoc* 7120; orange, ApcC interacting with Rep2 of *Synechococcus* 7002. **(c)** Superposition with ApcCs (Type II and III) of each cyanobacterium. Red dotted line indicates the loop regions (residues 9–26) of ApcC. Cyan, ApcC interacting with Rep3 of *T. vulcanus*; green, ApcC interacting with Rep2 of *Nostoc* 7120; orange, ApcC interacting with Rep2 of *Synechococcus* 7002. **(d)** Amino acid sequence alignment of ApcC subunit from *Thermosynechococcus vulcanus* (*T.*

vulcanus), *Nostoc* sp. PCC 7120 (*Nostoc* 7210), and *Synechococcus* sp. PCC 7002 (*Synechococcus* 7002).

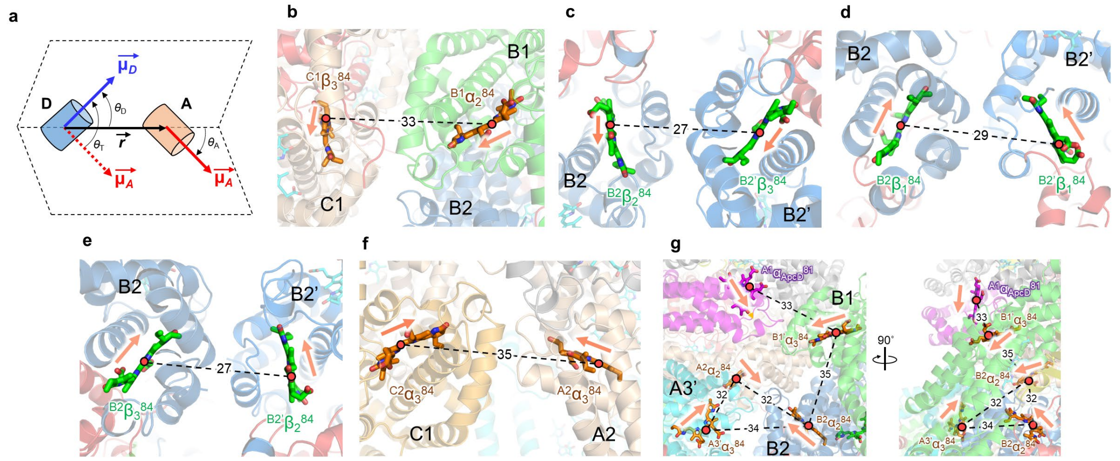

Supplementary Data Figure 11. Arrangement of chromophores between cylinders.

(a) Schematic model of the Förster resonance energy transfer. The transition dipole moment of the donor (d) and acceptor (a) are $\boldsymbol{\mu}_D$ and $\boldsymbol{\mu}_A$, respectively. **r** is the intermolecular center-to-center distance between D and A. θ_D is the angle between D–A connecting line and D transition dipole moment. θ_A is the angle between D–A connecting line and A transition dipole moments. θ_T is the angle between D and A transition dipole moment. **(b-g)** Chromophores in the B and C cylinders that may be associated with the energy transfer. The numbers on the dotted lines indicate the distances (Å) between the PCB pairs. The orange arrows indicate the direction of the transition dipole moment for each chromophore. The orientation factor, κ^2, is estimated using the following formula: $\kappa^2 = (\cos\theta_T - 3\cos\theta_D\cos\theta_A)^2$.

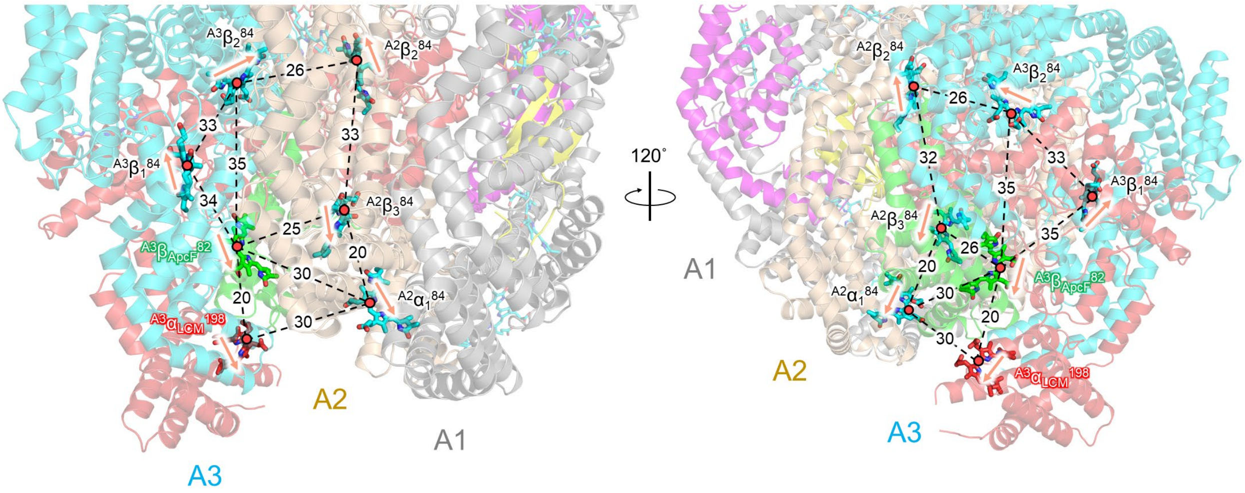

Supplementary Figure 12. Arrangement of chromophores around the terminal emitters. Chromophores in the A cylinders that may be associated with the energy transfer to the terminal emitters. The numbers on the dotted lines indicate the distances (Å) between the PCB pairs. The orange arrows indicate the direction of the transition dipole moment for each chromophore.

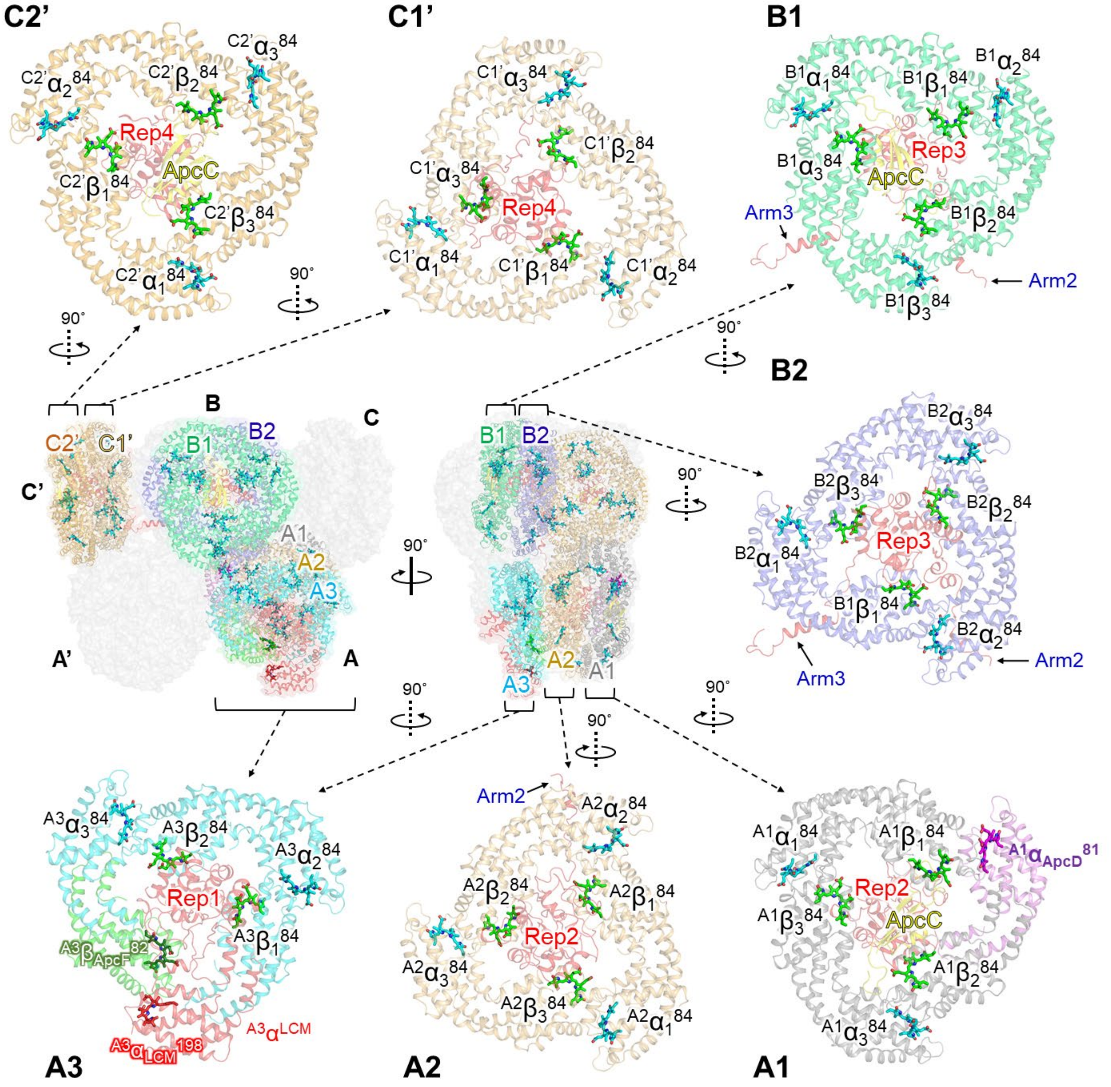

Supplementary Figure 13. Arrangement of chromophores in the PBS core and their names. PCBs bound in ApcA, cyan; PCBs bound in ApcB, green; PCBs bound in terminal emitters (ApcD, ApcE, and ApcF), magenta, red, and dark green, respectively.

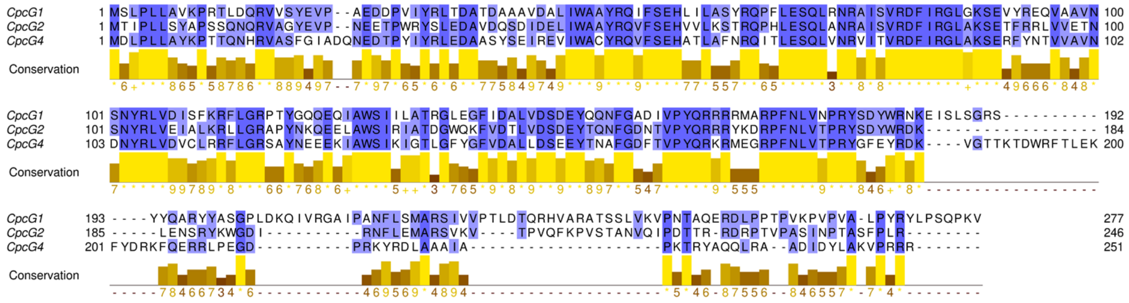

Supplementary Figure 14. Amino acid sequence alignment of CpcG1, CpcG2, and CpcG4 of *T. vulcanus*. The colors of the amino acid residues are indicated by percentage identities using Jalview.

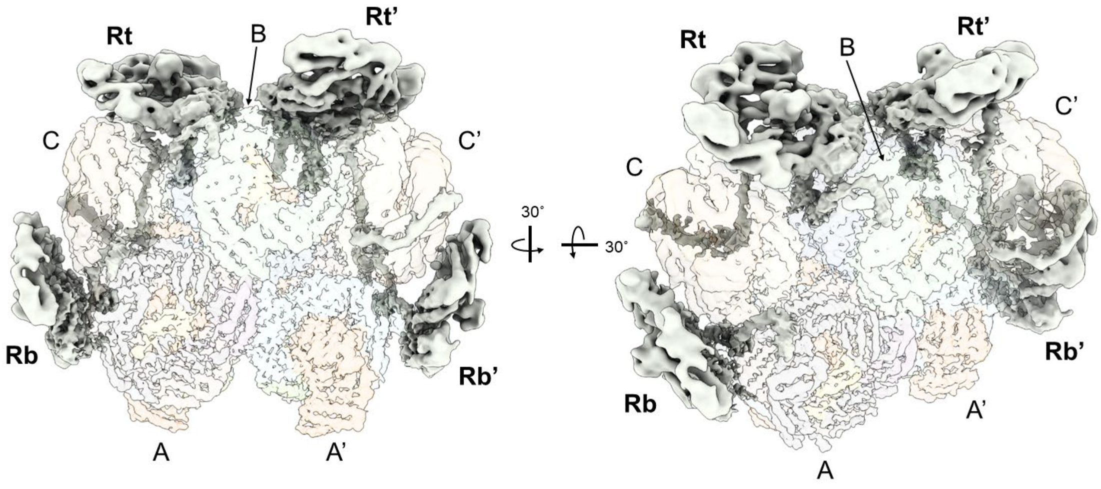

Supplementary Figure 15. The PC rods interacting with PBS core from *T. vulcanus*. The linker proteins extending from the PC rods (Rt, Rt', Rb, and Rb') interact with the A, B, and C cylinders of the PBS core. The PC rods and PBS core are shown in gray and translucent, respectively.

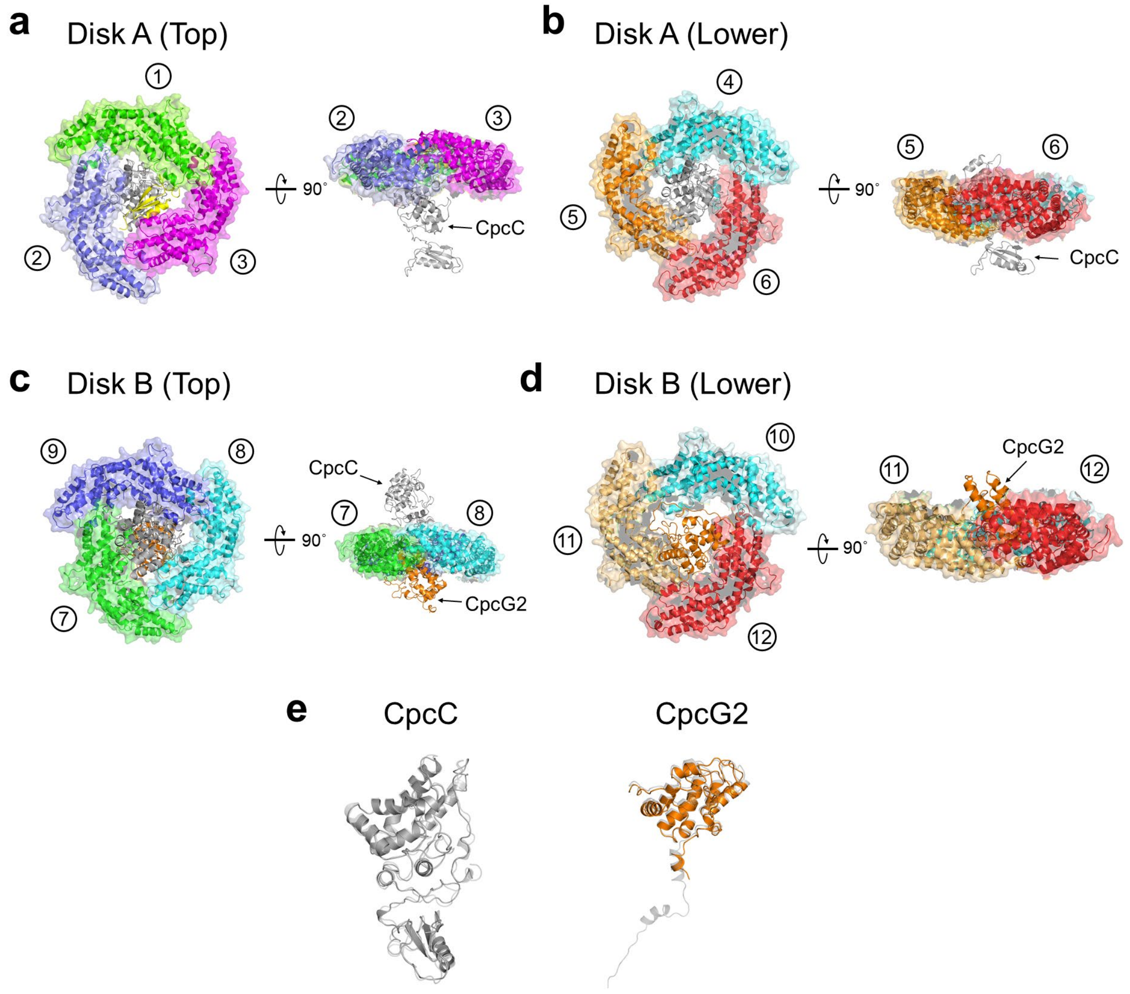

Supplementary Figure 16. Structural comparison of the PC rod from *T. vulcanus* (7EVA) and *Nostoc* 7120 (7EYD). (a–d) Superposition of each disk that comprises a PC rod. Cartoon helix and transparent surface models are PC rods from *T. vulcanus* and *Nostoc* 7120, respectively. **(E)** Superposition of linker proteins (CpcC and CpcG2). The cartoon helix and transparent helix models represent the PC rods from *T. vulcanus* and *Nostoc* 7120, respectively.

Supplementary Table 1. Statistics for data collection, processing, and refinement.

**Data collection**	PBS core (PDB: 7VEA, EMDB-31944)	PC rod (PDB: 7VEB, EMDB-31945)
Microscope	CRYO ARM 300	
Imaging device	K2 summit	
Accelerating voltage (kV)	300	
Imaging mode	Counting	
Data collection	JADAS	
Grid condition	Quantifoil R1.2/1.3 Cu 200 mesh Au sputtered	
Number of optics group	8	None
Nominal magnification	40,000×	
Total exposure time (sec)	6	
No. of frames	30	50
Total electron exposure ($e^-/Å^2$)	84.1	50.5
Defocus range (µm)	−0.5 – −1.5	
Original pixel size	1.24	
No. of total image sets	4,600	2,865
**Data processing**		
No. of used image sets	3,196	2,573
Initial particles (no.)	128,676	812,327
Final particles (no.)	25,532	159,537
Pixel size for final map	1.24	1.86
Symmetry imposed	C2	C1
Map resolution (Å)	3.72	4.19
B-factor for sharpening	−39.6	−89.3
FSC threshold	0.143	0.143
Map resolution range ($Å^2$)	3.28 – 20.73	4.02 – 6.96
**Refinement**		
Initial models (PDB code)	3O18, 3DBJ, 5Y6P, 6KGX	
Model resolution (Å)	3.76	4.24
FSC threshold	0.5	
Model composition		
Non-hydrogen atoms	111,542	35,543
Protein residues	15,594	4,536

Ligands	84	36
B factors (Å^2)		
Protein	144.6	119.5
Ligands	141.3	121.5
R.m.s. deviations		
Bond lengths (Å)	0.003	0.005
Bond angles (Å)	0.647	0.904
Validation		
MolProbity score	1.36	1.51
Clashscore	6.46	6.62
Poor rotamers (%)	0.58	0.68
Ramachandran plot		
Favored (%)	98.5	97.2
Allowed (%)	1.5	2.7
Disallowed (%)	0.0	0.1

Supplementary Table 2. Evaluation of the agreement between the cryo-EM map and the final model (PBS core).

Chain ID	Protein	Q-score*	Estimated resolution (Å)	Chain ID	Protein	Q-score*	Estimated resolution (Å)
aA, dA	ApcA	0.52	3.37	bQ, eQ	ApcA	0.48	3.62
aB, dB	ApcB	0.40	4.04	bR, eR	ApcB	0.53	3.34
aC, dC	ApcD	0.39	4.09	bS, eS	ApcA	0.40	4.05
aD, dD	ApcB	0.37	4.20	bT, eT	ApcB	0.46	3.71
aE, dE	ApcA	0.42	3.91	bU, eU	ApcA	0.49	3.52
aF, dF	ApcB	0.43	3.91	bV, eV	ApcB	0.43	3.86
aG, dG	ApcA	0.54	3.29	bW, eW	ApcA	0.46	3.72
aH, dH	ApcB	0.61	2.85	bX, eX	ApcB	0.45	3.78
aI, DI	ApcA	0.61	2.87	bY, eY	ApcC	0.55	3.20
aJ, dJ	ApcB	0.59	2.97	cA, fA	ApcA	0.41	3.98
aK, dK	ApcA	0.54	3.26	cB, fB	ApcB	0.41	3.98
aL, dL	ApcB	0.56	3.12	cC, fC	ApcA	0.40	4.03
aM, dM	ApcE	0.56	3.13	cD, fD	ApcB	0.51	3.42
aN, dN	ApcB	0.52	3.35	cE, fE	ApcA	0.50	3.51
aO, dO	ApcA	0.51	3.45	cF, fF	ApcB	0.49	3.52
aP, dP	ApcB	0.60	2.94	cG, fG	ApcA	0.36	4.30
aQ, dQ	ApcA	0.59	2.96	cH, fH	ApcB	0.37	4.22
aR, dR	ApcF	0.56	3.15	cI, fI	ApcA	0.40	4.06
aS, dS	ApcC	0.43	3.87	cJ, fJ	ApcB	0.44	3.81
bM, eM	ApcA	0.52	3.35	cK, fK	ApcA	0.48	3.61
bN, eN	ApcB	0.59	2.96	cL, fL	ApcB	0.39	4.08
bO, eO	ApcA	0.59	3.00	cM, fM	ApcC	0.51	3.46
bP, eP	ApcB	0.56	3.13	Total		0.49	3.55

*Q-score: An index showing resolvability of atoms, amino acid residues, and ligands assigned in a cryo-EM map (local resolution map). Resolution (Å) was estimated using the formula (Q-score = −0.1775× Resolution (Å) + 1.1192) according to Pintilie et al. (2020).

Supplementary Table 3. Evaluation of the agreement between the cryo-EM map and the final model (PC rod).

Chain ID	Protein	Q-score*	Estimated resolution (Å)
A	CpcA	0.42	3.95
B	CpcB	0.41	3.98
C	CpcA	0.39	4.12
D	CpcB	0.41	3.97
E	CpcA	0.42	3.91
F	CpcB	0.44	3.84
G	CpcA	0.42	3.93
H	CpcB	0.42	3.91
I	CpcA	0.40	4.04
J	CpcB	0.41	3.98
K	CpcA	0.41	3.99
L	CpcB	0.44	3.83
M	CpcA	0.42	3.97
N	CpcB	0.43	3.90
O	CpcA	0.36	4.26
P	CpcB	0.43	3.90
Q	CpcA	0.33	4.42
R	CpcB	0.36	4.30
S	CpcA	0.38	4.14
T	CpcB	0.33	4.42
U	CpcA	0.40	4.06
V	CpcB	0.42	3.94
W	CpcA	0.31	4.57
X	CpcB	0.35	4.33
Y	CpcD	0.48	3.60
Z	CpcC	0.47	3.64
a	CpcG2	0.41	4.01
Total		0.40	4.03

*Q-score: An index showing resolvability of atoms, amino acid residues, and ligands assigned in a cryo-EM map (local resolution map). Resolution (Å) was estimated using the formula (Q-score = −0.1775× Resolution (Å) + 1.1192) according to Pintilie et al. (2020).

Supplementary Table 4. Subunit proteins in the PBS core and PC rod of *T. vulcanus*.

Structure (PDB code)	Name	Protein	M. W. /kDa*	Number of molecules in a structural model	Remarks
PBS core (7VEA)	α subunit	ApcA	17.5	38	Main components of the PBS core
	β subunit	ApcB	17.4	40	Main components of the PBS core
	Core linker (L_C)	ApcC	7.9	6	Core-cap linker
	α subunit	ApcD	18.1	2	Terminal emitter
	Core-membrane linker (L_{CM})	ApcE	127.6	2	Terminal emitter. Components in the domain: α, Reps 1–4, and Arms 1–3.
	β subunit	ApcF	18.7	2	β-like subunit
PC rod (7VEB)	α subunit	CpcA	17.4	12	Main components of PC rod
	β subunit	CpcB	18.2	12	Main components of PC rod
	Rod linker (L_R)	CpcC	32.1	1	Phycocyanin-associated rod linker
	Rod-terminal linker (L_{RT})	CpcD	8.7	1	Rod-cap linker
	Rod-core linker (L_{RC})	CpcG1	31.6	0	Phycocyanin-associated rod-core linker
	Rod-core linker (L_{RC})	CpcG2	28.8	1	Phycocyanin-associated rod-core linker
	Rod-core linker (L_{RC})	CpcG4	29.6	0	Phycocyanin-associated rod-core linker

*The molecular weight (M. W.) of each subunit is estimated from the composition of the amino acid residues in each subunit.

Supplementary Table 5. Comparison of the structure and sequence identity of ApcE (L_{CM}) for each species.

	RMSD (Identity, %)*				
	ApcE (L_{CM})				
Name (PDB code)	αLCM	Rep1	Rep2	Rep3	Rep4
G. pacifica (5Y6P)	0.82 (58.6)	0.83 (62.0)	0.91 (52.7)	0.76 (60.2)	N/A
P. purpreum (6GKX)	0.76 (63.3)	0.71 (65.4)	0.76 (55.9)	0.73 (55.2)	N/A
Synechococcus (7EXT)	0.74 (61.3)	0.53 (82.9)	0.65 (72.6)	0.67 (74.0)	N/A
Nostoc (7EYD)	0.83 (75.3)	0.67 (85.9)	0.72 (81.8)	0.84 (74.0)	1.06 (77.0)

*Values in parentheses indicate sequence identity.

N/A: Not applicable.

Supplementary Table 6. Estimation of the orientation factor between major chromophores in the PBS core.

Pigment (D or A)	Pigment (A or D)	Orientation factor (κ^2)	Distance (Å)
${}^{C1}\beta_3{}^{84}$	${}^{B1}\alpha_2{}^{84}$	1.0	33
${}^{B2}\beta_2{}^{84}$	${}^{B2'}\beta_3{}^{84}$	0.8	27
${}^{B2}\beta_1{}^{84}$	${}^{B2'}\beta_1{}^{84}$	1.0	29
${}^{B2}\beta_3{}^{84}$	${}^{B2'}\beta_2{}^{84}$	0.8	27
${}^{C2}\alpha_3{}^{84}$	${}^{A2}\alpha_3{}^{84}$	3.0	35
${}^{A1}\alpha_{ApcD}{}^{81}$	${}^{A1}\beta_1{}^{84}$	2.8	21
${}^{B1'}\alpha_3{}^{84}$	${}^{B2}\alpha_2{}^{84}$	1.6	35
${}^{B2}\alpha_2{}^{84}$	${}^{A2}\alpha_2{}^{84}$	2.9	32
${}^{B2}\alpha_2{}^{84}$	${}^{A3'}\alpha_3{}^{84}$	2.5	34
${}^{A2}\alpha_2{}^{84}$	${}^{A3'}\alpha_3{}^{84}$	2.1	32
${}^{A3}\alpha_{LCM}{}^{198}$	${}^{A3}\beta_{ApcF}{}^{82}$	2.9	20
${}^{A3}\alpha_{LCM}{}^{198}$	${}^{A2}\alpha_1{}^{84}$	1.9	30
${}^{A3}\beta_{ApcF}{}^{82}$	${}^{A2}\alpha_1{}^{84}$	1.6	30
${}^{A3}\beta_{ApcF}{}^{82}$	${}^{A2}\beta_3{}^{84}$	1.3	25
${}^{A3}\beta_{ApcF}{}^{82}$	${}^{A3}\beta_2{}^{84}$	0.9	35
${}^{A3}\beta_{ApcF}{}^{82}$	${}^{A3}\beta_1{}^{84}$	1.9	34
${}^{A2}\alpha_1{}^{84}$	${}^{A2}\beta_3{}^{84}$	2.3	20
${}^{A3}\beta_2{}^{84}$	${}^{A2}\beta_3{}^{84}$	1.2	33
${}^{A2}\beta_2{}^{84}$	${}^{A3}\beta_2{}^{84}$	1.1	26
${}^{A3}\beta_1{}^{84}$	${}^{A3}\beta_2{}^{84}$	1.3	33

The distance (Å) between a pair of chromophores (donor (D) and acceptor (A)) and their orientation factor (κ^2).

Supplementary Table 7. Comparison of the structure and sequence identity of PC rod for each species.

		RMSD	
		T. vulcanus PC (3O2C)	*Nostoc* 7120 PC (7EYD)
PC monomer in PC rod	1	0.55	0.78
	2	4.06	3.36
	3	2.60	2.63
	4	0.76	0.78
	5	1.49	0.73
	6	1.40	1.02
	7	21.3	0.79
	8	14.8	1.10
	9	10.6	0.87
	10	11.6	2.79
	11	18.3	1.04
	12	15.4	1.36
